# Homotype-Targeted Biogenic Nanoparticles to Kill Multidrug-Resistant Cancer Cells

**DOI:** 10.3390/pharmaceutics12100950

**Published:** 2020-10-09

**Authors:** Imran Shair Mohammad, Birendra Chaurasiya, Xuan Yang, Chuchu Lin, Hehui Rong, Wei He

**Affiliations:** 1 Department of Pharmaceutics, School of Pharmacy, China Pharmaceutical University, Nanjing 211198, China; shair@mail.sysu.edu.cn; 2 School of Pharmaceutical Sciences, Sun Yat-sen University, University Town, Guangzhou 510006, China; yangx287@mail2.sysu.edu.cn (X.Y.); linchch3@mail2.sysu.edu.cn (C.L.); ronghh3@mail2.sysu.edu.cn (H.R.); 3 Department of Pediatrics, Division of Critical Care, Northwestern University, Feinberg School of Medicine, Chicago, IL 60611, USA; birendra.chaurasiya@northwestern.edu

**Keywords:** multidrug resistance, P-gp, biogenic nanoparticles, homotypic binding, apoptosis

## Abstract

“Off-targeting” and receptor density expressed at the target sites always compromise the efficacy of the nanoparticle-based drug delivery systems. In this study, we isolated different cell membranes and constructed cell membrane-cloaked biogenic nanoparticles for co-delivery of antitumor paclitaxel (PTX) and multidrug resistance (MDR)-modulator disulfiram (DSF). Consequently, MDR cancer cell membrane (A549/T)-coated hybrid nanoparticles (A549/T CM-HNPs) selectively recognized the source cells and increased the uptake by ninefold via the homotypic binding mechanism. Moreover, the A549/T CM-HNPs sensitized MDR cells to PTX by suppressing P-glycoprotein (P-gp) activity by 3.2-fold and induced effective apoptosis (70%) in homologous A549/T cells. Cell-membrane coating based on the “homotypic binding” is promising in terms of promoting the accumulation of chemotherapeutics in MDR cells and killing them.

## 1. Introduction

Cancer is a leading cause of death worldwide, due to the multiple carcinogenic processes entailed with various incomprehensible and complex cellular pathways [1,2]. Despite the advancement in conventional cancer treatments (chemotherapy, radiation, or surgery), cancer therapy is still hampered by poor specificity, inadequate drug distribution, and serious systemic toxicities [3]. Nanoparticle-mediated drug delivery system (NDDS) improves drug accumulation in tumors and allows for enhanced antitumor efficacy. However, “off-targeting” and receptor density expressed at the target sites always compromise the efficacy of the NDDS. Recently, in order to accurately target tumor cells, various bio-mimetic strategies were explored to deliver payload at intended sites [4,5]. Coating nanoparticles (NPs) with source cell membrane-derived from the homologous tumor enabled NP self-recognition, enhancing internalization and efficient tumor homing to the homologous tumors [6]. Intriguingly, the cancer cell membrane-coated NPs are capable to evade “immune-surveillance” using combination of cell surface signals and secreted factors [7,8].

Nanocrystals are carrier-free solid drug particles with a diameter of less than 1000 nm and with crystalline characteristics. Nanocrystals are promising to treat various type of diseases, owing to their high drug loading (as high as 100%), reduced personal variability and food effects, improved treatment outcomes with decreased side effects, and low toxicity to normal organs; thus, nanocrystals represent promising nanomedicines [9,10]. Recently, combination therapy has been employed as a primary cancer treatment in clinics. Combining multiple drugs with different molecular targets not only reduces the cancer cell progression but also functions synergistically or additively for improved therapeutic efficacy at minimum systemic toxicity [11]. However, the treatment efficacy of combinatorial formulations is always discounted by the development of multidrug resistance (MDR). The MDR in cancer is a multifactorial response of numerous independent as well as interdependent cellular pathways [12], wherein the most critical is the activation of efflux transporter (P-glycoprotein (P-gp), a product of the MDR-1 gene), which results in unpredictable therapeutic effects due to the decreased accumulation of drugs inside the cells [13]. In order to reverse MDR, different MDR inhibitors such as curcumin, disulfiram (DSF), calmodulin antagonist, ginseng, ginsenoside Rh2, and adriamycin have been delivered in combination with antitumor drugs to treat MDR tumors [14].

**Scheme 1:**
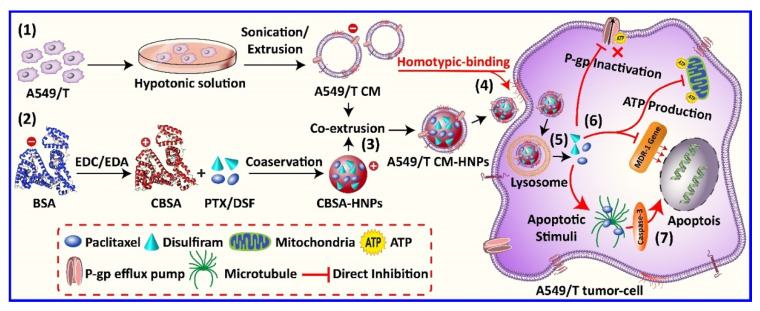
(**1**) The A549/T cells were suspended in hypotonic lysis buffer supplemented with protease inhibitor cocktail followed by homogenization, series of centrifugation, and sonication to produce A549/T cell membrane vesicles. (**2**) The cationic bovine serum albumin (CBSA) was synthesized from native bovine serum albumin (BSA) by ethylenediamine modification reaction, and CBSA hybrid nanoparticles (CBSA-HNPs) were prepared by conservation method. (**3**) A549/T cell membrane-coated hybrid nanoparticles (A549/T CM-HNPs) were prepared by co-incubating cell membrane vesicles and hybrid nanoparticle core, followed by sonication and co-extrusion through a 200 nm polycarbonate membrane. (**4**) A549/T CM-HNPs targeted homologous A549/T cells via homotypic-binding mechanism, releasing (**5**) paclitaxel (PTX) and disulfiram (DSF) into the cytosol. (**6**) The multidrug resistance (MDR) modulator DSF inhibited the ATP concentration, downregulated the MDR-1 gene, and therefore suppressed P-glycoprotein (P-gp) and increased intracellular PTX concentration, thus inducing (**7**) caspase-3-dependent MDR tumor cell (A549/T) apoptosis.

In our previous studies, paclitaxel–DSF hybrid nanocrystals were prepared and improved antitumor response in MDR tumor (A549/T) was demonstrated [10,15]. Nonetheless, the hybrid nanoparticles had modest targeting ability to MDR cancer cells due to poor recognition by the cells. In this study, by taking advantage of homotypic binding, we fabricated various cell membrane-camouflaged hybrid biogenic nanoparticles of paclitaxel (PTX) and DSF to target A549/T cells. The MDR tumor cell membrane-coated hybrid NPs (A549/T CM-HNPs) demonstrated excellent self-recognition and targetability to the homologous A549/T cells when compared to the heterologous cell membrane-coated nanoparticles (red blood cell (RBC) CM-HNPs, human hepatic normal cell line (LO2) CM-HNPs, and murine mammary carcinoma cell line (4T1) CM-HNPs). Finally, the A549/T CM-HNPs significantly delivered PTX and DSF to the MDR tumor cells and induced potent apoptosis by inactivating P-gp-associated drug efflux pump to relinquish MDR (Scheme 1).

## 2. Materials and Methods

### 2.1. Materials, Cell Culture, and Animals

Paclitaxel (PTX, ≥99% purity) was purchased from Yew Biotechnology Co., Ltd. (Suzhou, Jiangsu, China). Disulfiram (DSF, ≥97% purity), bovine serum albumin (BSA, ≥98% purity), fluorescein 5(6)-isothiocyanate (FITC, 90% purity), and 3-(4,5-dimethylthiazol-2yl)-2,5-diphenyltetrazolium bromide (MTT, 98% purity) were purchased from Sigma-Aldrich Co., Ltd. (St. Louis, MO, USA). The annexin V-FITC/PI apoptosis detection kit was obtained from Nanjing KeyGen Biotech Co., Ltd. (Nanjing, China); the caspase-3 and -9 calorimetric assay kit was obtained from Nanjing KeyGen Biotech Co., Ltd. (Nanjing, China); the ATP assay kit was obtained from Solarbio Science and Technology Co., Ltd. (Beijing, China), and the bicinchoninic acid (BCA) protein estimation kit was purchased from Beyotime Institute of Biotechnology Co., Ltd. (Haimen, China). Human lung adenocarcinoma cells (A549) were purchased from Nanjing KeyGen Biotech Co., Ltd. (Nanjing, China) and Taxol-resistant A549/T cells were established in our lab by dose escalation method. Fetal bovine serum (FBS) and RPMI-1640 cell culture medium were obtained from Wisent Inc. (St. Bruno, QC, Canada). PBS, trypsin-EDTA (more than 6000 U/mg), and a 100 U/mL penicillin + 100 μg/mL streptomycin solution were obtained from Nanjing KeyGen Biotech Co., Ltd. (Nanjing, China).

Taxol sensitive human lung adenocarcinoma cell line (A549) and Taxol-resistant human lung adenocarcinoma cell line (A549/T) were cultured in RPMI-1640 cell culture medium supplemented with 10% FBS and 100 U/mL penicillin + 100 μg/mL streptomycin at 37 °C in a 5% CO_2_-humidified incubator (Thermo Fisher, USA). To get the whole blood, the adult Sprague-Dawley (SD) rat (200 ± 20 g) and female BALB/c nude mice (15–17 g) were purchased from Nanjing Qinlong animal center (Nanjing, China). The animals were kept in a pathogen-free environment at a temperature of 22 ± 2 °C and were given free access to food and water. The blood collection experiment was approved by the Institutional of Animal Care and Use Committee at China Pharmaceutical University, and followed local, national, ethical, and regulatory principles.

### 2.2. Methods

#### 2.2.1. Synthesis and Characterization of Cationic BSA

Cationic BSA (CBSA) was synthesized from native BSA by ethylenediamine modification reaction, as described previously [16]. Briefly, 5 mL of 20% (w/v) BSA solution was slowly added to 125 mL of ethylenediamine solution (0.9 M, pH 4.75) under continuous stirring. Subsequently, 200 mg of 1-Ethyl-3-(3-dimethylaminopropyl)-carbodiimide hydrochloride (EDC) was introduced, and the mixture was stirred at room temperature for 2 h. The reaction was stopped by addition of 650 μL acetate buffer (4 M, pH 4.75). Next, the reaction solution was loaded in a protein concentrator (Molecular weight cut-off (MWCO)-30 kDa) sample chamber (Thermo Fisher, USA), tightly caped and centrifuged at 4700× *g* for 15 min at 4 °C until volume was reduced up to 10 mL. Next, the retentate was gently aspirated, collected from sample chamber, and dialyzed against distilled water by using a regenerated cellulose dialysis bag of MWCO-3.5 kDa for 72 h. The total CBSA recovery was calculated about 85% of initial protein concentration. Finally, the CBSA concentrate was freeze-dried to obtain CBSA. After synthesis, the native BSA and CBSA were characterized by determining their zeta potentials by using a ZetaPlus Zeta Potential Analyzer (Brookhaven Instruments, Holtsville, NY, USA) at 25 °C and SDS-PAGE assay by taking native BSA as control.

#### 2.2.2. Isolation of Cell Plasma Membranes

The cell plasma membrane of RBCs, LO2, 4T1, and A549/T was isolated by using a previously described protocol with few modifications [17]. Briefly, the RBCs were collected from whole blood of female BALB/c nude mice from the orbit of mice with the addition of 1.5 mg of EDTA per milliliter of blood for anticoagulation, while LO2, 4T1, and A549/T cells were harvested and washed with PBS three times. Next, the cells were resuspended in hypotonic lysis buffer supplemented with protease inhibitor cocktail (MedChem Express LLC, USA) on ice for 5 min. Thereafter, individual cells were homogenized using a Dounce homogenizer with a pestle. To remove the unbroken cells and cellular nuclei, we centrifuged the homogenate at 800× *g* for 10 min at 4 °C. Next, the supernatant was centrifuged at 10,000× *g* for 15 min to remove the cell mitochondria, followed by centrifugation at 100,000× *g*, 4 °C for 1 h. Then, the supernatant was discarded, and the pellet was washed with 10 mM Tris-HCl and 0.5 mM EDTA with protease inhibitor cocktail and freeze-dried, weighted, and stored at −80 °C.

#### 2.2.3. Cell Membrane Vesicle Preparation and Characterization

The collected cell membranes (RBCs, LO2, 4T1, A549/T) were sonicated in a capped glass vial by using a bath sonicator at 42 kHz and power of 100 W for 5 min. The resulting individual cell type vesicles were then physically extruded for 11 passes through 400 nm polycarbonate porous membranes using an Avanti mini extruder (Avanti Polar Lipids, AL, USA) to obtain cell membrane vesicles (RBC CM, LO2 CM, 4T1 CM, A549/T CM). Finally, the size, polydispersity index (PDI), and zeta potentials of RBC CM, LO2 CM, 4T1 CM, and A549/T CM were monitored using a ZetaPlus Zeta Potential Analyzer (Brookhaven Instruments, Holtsville, NY, USA) at 25 °C. 

The biocompatibility of BSA, CBSA, RBC CM, LO2 CM, 4T1 CM, and A549/T CM was determined by using an MTT assay [18]. Briefly, A549 and A549/T cells were seeded in a 96-well plate (1 × 10^4^ cells per well) and cultured for 24 h to allow the cells to attach to the surface. Next, the cells were incubated with different concentrations of BSA; CBSA; and RBC CM, LO2 CM, 4T1 CM, and A549/T CM for 24 h. For this experiment, the untreated cells were used as a control. Next, 20 μL of MTT solution in PBS (5 mg/mL) was added to each well and incubated for 4 h at 37 °C, followed by the addition of 150 μL of DMSO. Finally, the absorbance was determined at 490 nm by using a spectrophotometer (Thermo Scientific Multiskan FC Microplate Photometer, Waltham, MA, USA).

#### 2.2.4. Preparation of Nanoparticle Cores

PTX- and DSF-loaded cationic bovine serum albumin (CBSA) hybrid nanoparticle cores (CBSA-HNPs) were prepared by simple coacervation method. Briefly, CBSA was dissolved into 4 mL of water, and the pH of the protein solution was adjusted to 7 and filtered through a 0.45 µm filter, followed by mixing for 10 min at 1000× *g* with a magnetic stirrer at room temperature. Next, PTX (6 mg) and DSF (1 mg) were dissolved into 2 mL of ethanol and continuously added at 1 mL/min. Subsequently, the extra ethanol was continuously added at the same flow rate until the solution appeared milky. Next, under dimmed light, we added 200 µL of 4% glutaraldehyde and the mixture was placed for 6 h at 25 °C for crosslinking. Finally, ethanol was evaporated on a rotary evaporator at 40 °C under reduced pressure, followed by centrifugation at 12,000× *g* for 15 min at 25 °C, and supernatant was collected to determine the drug loading (DL) and entrapment efficacy (EE) of PTX and DSF, respectively. 

#### 2.2.5. Functionalization of Nanoparticle Cores

The RBCs, LO2, 4T1, and A549/T cell membrane-coated hybrid nanoparticles (RBC CM-HNPs, LO2 CM-HNPs, 4T1 CM-HNPs, and A549/T CM-HNPs) were prepared by co-incubating respective cell membrane vesicles and hybrid nanoparticle cores followed by sonication and co-extrusion through a 200 nm polycarbonate membrane. Finally, RBC CM-HNPs, LO2 CM-HNPs, 4T1 CM-HNPs, and A549/T CM-HNPs (CM-HNPs) were centrifuged at 500× *g* for 3 min to separate any precipitates formed during the extrusion process.

The BSA- and FITC-labelled hybrid nanoparticles were prepared by the same procedure, except BSA was used in place of CBA to prepare BSA hybrid nanoparticles (HNPs), while FITC was co-dissolved with drug solution (FITC, PTX, DSF) to prepare FITC-loaded hybrid nanoparticles (FITC-HNPs, FITC-RBCs CM-HNPs, FITC-LO2 CM-HNPs, FITC-4T1 CM-HNPs, FITC-A549/T CM-HNPs).

#### 2.2.6. Determination of Membrane-Associated Protein 

The presence of membrane-associated proteins on RBC CM, LO2 CM, 4T1 CM, and A549/T CM (cell membrane vesicles) as well as on CM-HNPs was determined by Coomassie blue staining assay. The RBC CM, LO2 CM, 4T1 CM, A549/T CM, and CM-HNPs were collected after 0, 24, and 48 h. Next, the prehomogenated cell membrane vesicles and respective CM-HNPs were lysed in radioimmunoprecipitation (RIPA) lysis buffer (50 mM Tris, 150 mM NaCl, 1% Triton X-100, 1% sodium deoxycholate, 0.1% SDS) with protease inhibitor solution on ice for 5 min. Next, lysates were centrifuged at 13,000× *g* for 5 min at 4 °C to collect the supernatants and subjected to BCA protein assay (Beyotime Biotechnology, Haimen, China) for total protein quantification. Thereafter, the individual supernatant was mixed with SDS loading buffer and heated up to 95 °C for 5 min. A 20 μg equivalent protein for each sample was added in each well of 10% SDS-PAGE in an electrophoresis chamber system (Bio-Rad Laboratories, Philadelphia, PA, USA) and run at 120 V for 1.5 h. Finally, the gel was stained with Coomassie blue (Beyotime Biotechnology, Haimen, China) overnight, followed by distaining, and the images were recorded.

#### 2.2.7. Particle Size, Zeta Potential, and TEM Examination

The particle size, PDI, and zeta potentials (*n* = 3) of the nanoparticles were determined after diluting up to 10 vol.% by using ZetaPlus (Brookhaven Instruments, Holtsville, NY, USA) according to the dynamic light scattering (DLS) principle at 25 °C, where the acquisition lag time of each measurement was 300 s and auto slope analysis was selected for baseline normalization. The TEM examination of A549/T CM and A549/T CM-HNPs was conducted by diluting the samples 200 times. The samples at 1 mg/mL were placed on copper mesh for 1 min and then negatively stained with three drops of 1% uranyl acetate. The excess solution was removed with filter paper and imaged on JEM-1230TEM (Tokyo, Japan) at an accelerated voltage of 200 kV. 

#### 2.2.8. In Vitro Drug Release Kinetics

The in vitro drug release kinetics of different formulations was determined by dialysis method as described in our previous report [10]. Briefly, the dialysis was performed by using a regenerated cellulose dialysis bag of MWCO-3.5 kDa in an incubator with a shaking speed of 100 rpm/min at 37 °C. The release media was PBS with 1% (w/v) Tween-80 at pH 7.4. At predetermined time intervals, we withdrew 2 mL of sample from the dialysis bag and replenished it with fresh release medium. Upon centrifugation at 15,000× *g* for 5 min, the concentrations of PTX and DSF in the supernatant were quantified using an HPLC system equipped with an LC-10AT pump and SPD10A UV-detector (Shimadzu, Tokyo, Japan). PTX and DSF were separated on a Diamonsil C-18 column (4.6 mm × 250 mm) at 227 and 275 nm, respectively. A mixture of methanol and water (80:20, v/v) was used as mobile phase at a flow rate of 1.0 mL/min at 37 °C, with the injection volume being 20 μL.

#### 2.2.9. Membrane Coating Optimization and Stability Studies

To optimize the membrane/core ratio, different CM-HNPs were prepared with increasing amount of respective cell membrane/core ratios (w/w) ranging from 0.5–2 mg at a fixed CBSA concentration of 1 mg/mL for 4 h at 4 °C. In this experiment BSA HNP core with no membrane was used as control. The particle size was measured right after the preparation and after 7 days of storage in 1x PBS by using ZetaPlus (Brookhaven Instruments, Holtsville, NY, USA) at 25 °C. For the stability experiment, at various pre-determined time points, we carried out CM-HNPs in 1xPBS + 30% serum at pH 7.4 to measure nanoparticle size by using ZetaPlus (Brookhaven Instruments, Holtsville, NY, USA) at 25 °C.

#### 2.2.10. In Vitro Antitumor Assay

The cytotoxicity of CM-HNPs was assessed using MTT assay. Briefly, A549 and A549/T cells were seeded in a 96-well plate (4 × 10^4^ cells per well) and incubated for 24 h. Next, the cells were treated with different formulations with increasing concentration (0.1, 0.5, 1, 5, 10, 15, 20, 25, 50, 10 µg/mL) for 24 h. In this experiment, the untreated cells were used as the control. Thereafter, 20 μL of MTT solution (5 mg/mL) was added into each well and incubated at 37 °C for 4 h, followed by the addition of 150 μL of DMSO. Finally, the absorbance was determined by using MTT reader at 570 nm (Thermo Scientific, USA).

#### 2.2.11. Homotypic Uptake Study 

For cell uptake study, the A549 and A549/T cells were seeded in 24-well plates at a density of 1 × 10^5^ cells per well and cultured for 24 h at 37 °C. Next, the cells were incubated with FITC-labelled formulations for 0.5, 1, 2, 4, 8, and 12 h at 37 °C at a fixed FITC concentration of 50 ng/mL in PBS. Next, the cells were collected, washed three times with PBS, and resuspended in 200 μL of PBS for flow cytometry analysis (FACSCalibur, BD Biosciences, San Jose, CA, USA).

For cell uptake qualitative image analysis, the A549 and A549/T cells (1 × 10^4^ cells) were cultured in 6-well plates for 24 h at 37 °C. Next, the cells were incubated with FITC-A549/T CM-HNPs at a fixed FITC concentration of 50 ng/mL for 0.5, 1, 2, 4, 8, and 12 h. The cells were washed with PBS five times to quench extracellular fluorescence and were observed under a fluorescence microscope (Olympus Life Science Solutions, USA).

#### 2.2.12. Apoptosis and Caspase Assay

For apoptosis assay, A549 and A549/T cells were cultured on a 6-well plate at 5 × 10^5^ cells per well for 24 h. First, the cells were treated with different formulations for 24 h, washed with PBS three times, incubated with annexin V-FITC/PI for apoptosis detection at room temperature for 15 min in dark, and assayed by flow cytometry. Furthermore, to observe the cellular and nuclei morphology, we treated cells with A549/T CM-HNPs at a fixed PTX concentration of 5 µg/mL for 24 h and stained them by 4′,6-diamidino-2-phenylindole (DAPI) for 5 min at 37 °C. The cellular and nuclear morphology was visualized under fluorescence microscope (Olympus Life Science Solutions, USA).

The underlying mechanism of apoptosis was studied by employing caspase-3 assay kits. Briefly, cells were treated with different NPs for 24 h, washed two times with PBS, and collected, followed by the addition of 200 μL of lysate buffer for the caspase-3 assay and 100 μL for the caspase-9 assay and incubation for 20 min on ice. Next, the cell lysate was collected by centrifugation at 10,000× *g* at 4 °C. At this point, the total protein concentration was determined by using a BCA protein assay kit. Thereafter, the 50 μL of cell lysate was mixed with 50 μL of 2× reaction buffers and 5 μL of caspase substrate and incubated at 37 °C for 4 h in the dark. Finally, the optical density of reaction mixture was determined at 405 nm by a spectrophotometer (Thermo Scientific, Waltham, MA, USA).

#### 2.2.13. P-gp Drug Efflux Inhibition and Intracellular ATP Determination 

The A549/T cells (1 × 10^5^ cells per well) were incubated for 4 h with the free RH-123 (5 mM) in the presence of A549/T CM-HNPs and A549/T CM-PTXNPs alone. Next, the cells were collected and washed with cold PBS, and then fluorescence intensity was measured by flow cytometry. Furthermore, A549/T cells (1 × 10^4^ cells per well) were cultured in 6-well plates. After 24 h, cells were co-incubated with free RH-123 (5 mM), A549/T CM-HNPs, and A549/T CM-PTXNPs for 1, 2, and 4 h and observed under a fluorescence microscope (Olympus Life Science Solutions, Waltham, MA, USA). 

The intracellular ATP concentration was determined as described in our previous report [15]. Briefly, A549/T cells at a density of (1 × 10^5^ cells per well) were seeded in 6-well plates for 24 h and incubated with A549/T CM-HNPs and A549/T CM-PTXNPs at 37 °C for 4 h. After 4 h, cells were lysed by using lysing solution (200 µL) and centrifuged at 12,000× *g* for 5 min at 4 °C. Thereafter, 20 µL of supernatant of individual sample was mixed with ATP-testing solution (100 µL) on a black 96-well plate for 10 min. Finally, the luminescence intensity was subsequently determined by luminoskan microplate luminometer (Thermo Scientific, Waltham, MA, USA).

#### 2.2.14. Hemotoxicity Assay

The hemotoxicity of A549/T CM-HNPs was determined by co-incubating A549/T CM-HNPs with red blood cells (RBCs), and hemolytic activity was recorded. Briefly, the rat blood was collected and fibrinogen was removed by stirring for 10 min. Next, blood was diluted (10 times), centrifuged at 1500× *g* for 10 min to collect RBC pallet, and resuspended in saline to adjust the concentration of 2% (v/v). Next, RBC suspension was incubated with A549/T CM-HNPs at a series of PTX concentration (0.5, 1, 1.5, 2, 2.5 mg/mL) in an Eppendorf tube (1.5 mL) for 2 h at 37 °C, followed by centrifugation (8000× *g*) for 10 min, wherein intact RBCs were collected. In this experiment, Triton-X 100 (20%) and saline were used as positive and negative controls (PC and NC), respectively. Finally, the amount of hemoglobin released was calculated by taking the absorbance of supernatant at 540 nm. The percent hemolysis was determined as follows:

(1) $$\mathrm{Hemolysis}\;\left(\%\right)=\frac{\left(A_{sample}-A_{b}-A_{0}\right)}{\left(A_{100}-A_{0}\right)}\;\times 100$$

where *A_sample_* is the absorbance of sample (β-LG), *A*_b_ is the absorbance of blank sample, *A*_0_ is the absorbance of 0% hemolysis (saline), and *A*_100_ is the absorbance of 100% hemolysis (Triton-X 100).

Moreover, to visualize the extent of RBC membrane damage, after incubation with A549/T CM- HNPs at a series of PTX concentration (0.5, 1, 1.5, 2, 2.5 mg/mL) in an Eppendorf tube (1.5 mL) for 2 h at 37 °C, the RBCs were collected by centrifuging at 1500× *g* for 10 min. At this point, the treated RBCs were resuspended in saline (1 mL) and further diluted (1:10). Finally, 100 µL of diluted samples were mounted on a glass slide and observed under bright field (Olympus Life Science Solutions, Waltham, MA, USA).

#### 2.2.15. Statistical Analysis

The results are described as means ± SD, and each value was the mean of three-replicate independent experiments. The statistical analysis was obtained by applying a paired *t*-test, one-way or two-way ANOVA, and the significance difference is indicated as * *p* < 0.05, ** *p* < 0.01, or *** *p* < 0.001.

## 3. Results and Discussion 

### 3.1. Preparation and Characterization of Cell Membrane-Coated Hybrid Biogenic Nanoparticles

**Figure 1:**
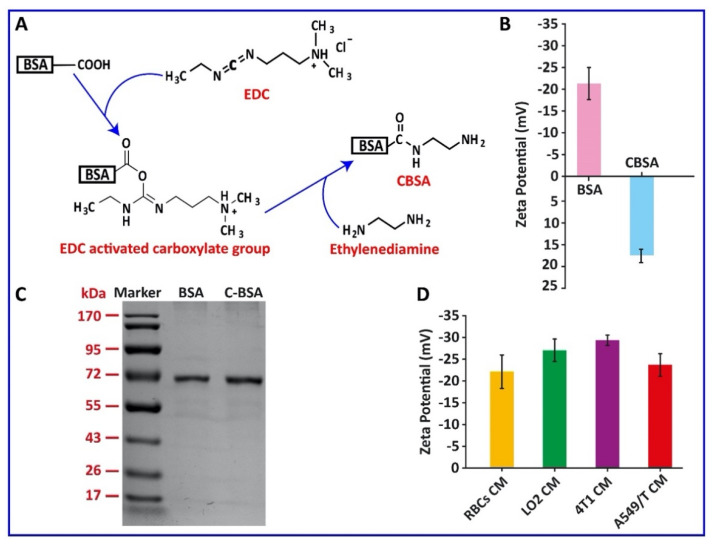
Synthesis and characterization of CBSA and different cell membrane vesicles. (**A**) Synthesis scheme of CBSA by ethylenediamine modification, (**B**) surface charge of native and CBSA, (**C**) SDS-PAGE of native BSA and CBSA, (**D**) surface charge of different cell membrane vesicles.

**Figure 2:**
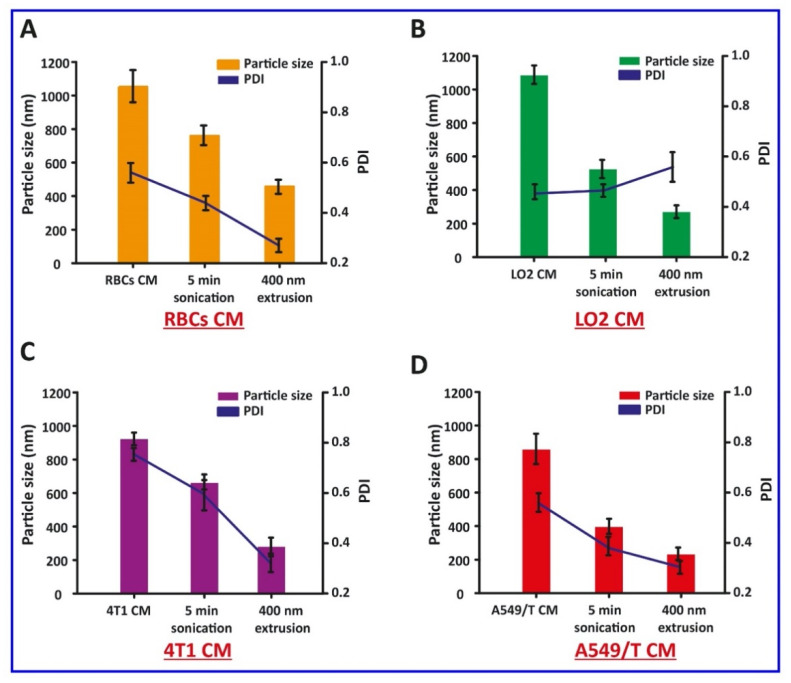
Isolation, preparation, particle size, and PDI of cell membrane vesicles. (**A**) Red blood cells (RBCs), (**B**) LO2, (**C**) 4T1, and (**D**) A549/T.

Here, the preparation of CM-HNPs was accomplished in three major steps, (1) synthesis of CBSA; (2) preparation of RBCs, LO2, 4T1, and A549/T CM vesicles; and (3) cell membrane cloaking of CBSA-HNP cores. The CBSA was synthesized from native BSA through ethylenediamine modification by amide linkages, and zeta potential measurement of BSA and CBSA showed −21 mV and +17 mV, respectively. Furthermore, the SDS-PAGE assay revealed that the molecular weight of CBSA did not change when compared to the native BSA, i.e., approximately 68 kDa (Figure 1A–C). Next, the respective cell membrane was collected by emptying the intracellular contents with a combined procedure of hypotonic cytolysis and differential centrifugation [19]. The individual cell membrane vesicles were prepared by mechanical extrusion from 400 nm polycarbonate porous membrane, and after sonication for 5 min, we monitored their size (Figure 2A–D). Moreover, the zeta potential values of RBC CM, LO2 CM, 4T1 CM, and A549/T CM were −22.6, −27.1, −28.6, and −24.4 mV, respectively (Figure 1D).

**Figure 3:**
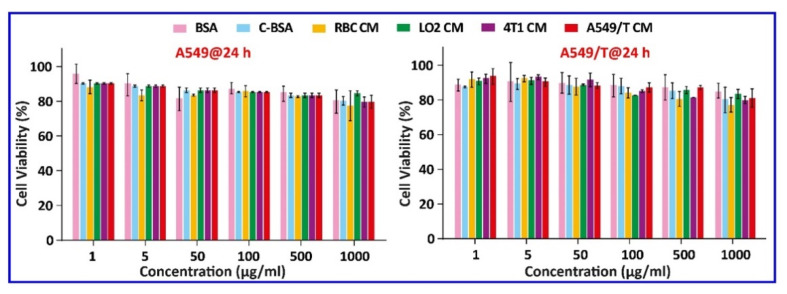
Biocompatibility investigation in A549 or A549/T cells after treatment for 24 h at concentrations that varied from 1 to 1000 µg/mL; *n* = 3.

**Figure 4:**
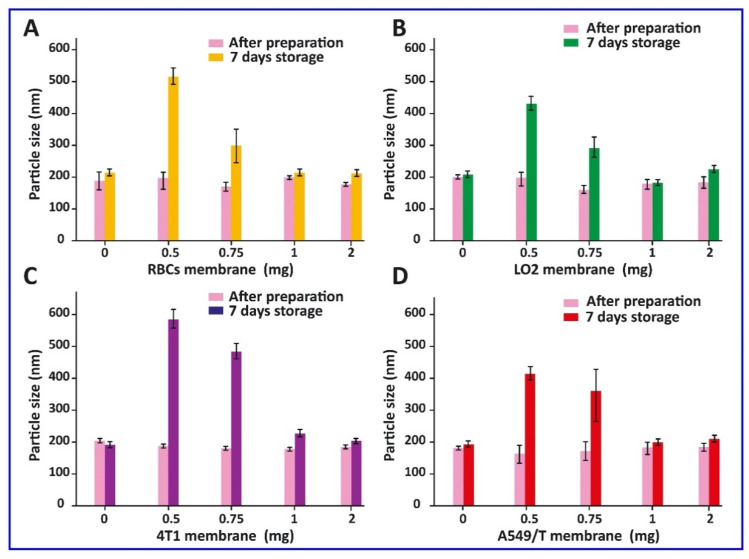
Membrane-coating optimization. Hydrodynamic size of (**A**) RBC CM-HNPs, (**B**) LO2 CM-HNPs, (**C**) 4T1 CM-HNPs, and (**D**) A549/T CM-HNPs at various weight ratios of membrane protein and nanoparticle after synthesis and after 7 days storage in PBS (pH 7.4); *n* = 3.

**Figure 5:**
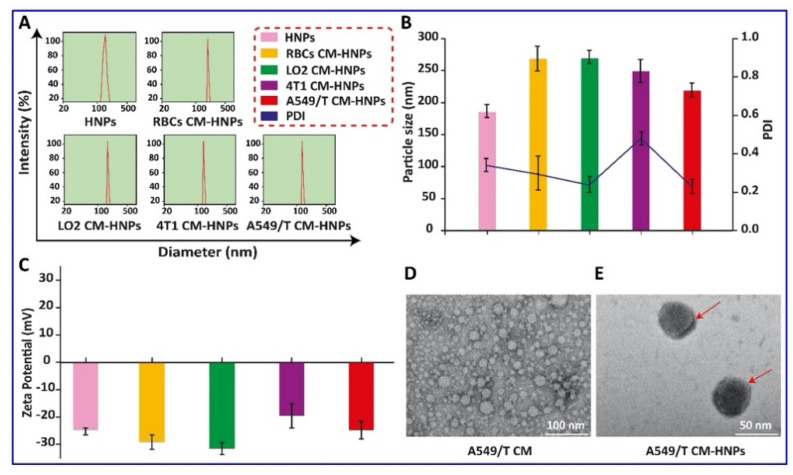
Preparation and characterization of biogenic HNPs. (**A**,**B**) Particle size and PDI, (**C**) surface charge, (**D**,**E**) TEM images.

**Figure 6:**
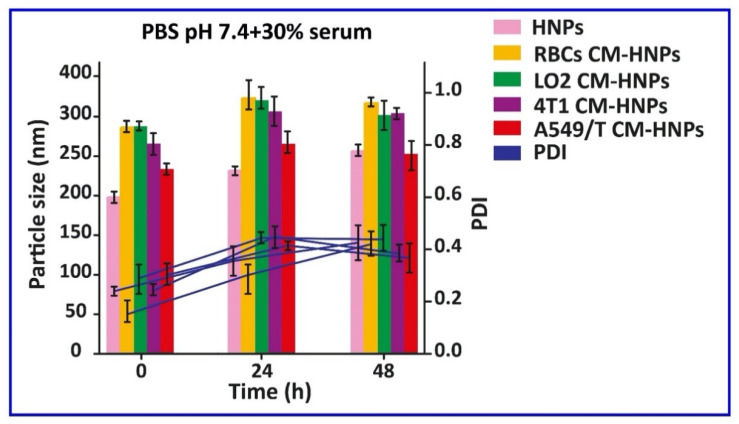
Stability HNPs, RBC CM-HNPs, LO2 CM-HNPs, 4T1 CM-HNPs, and A549/T CM-HNPs in PBS (pH 7.4) + 30% FBS over a span of 48 h.

The biocompatibility of these vesicles was studied by incubating them with A549 and A549/T cells with increasing concentration up to 1000 µg/mL for 24 h. The results indicated that none of these induced serious cellular toxicities and the cell viability was above 80% at all concentrations, which ruled out the material-associated toxicity (Figure 3). Consequently, these materials were biocompatible and could be employed for the fabrication of nanomedicine. Next, we prepared biogenic HNPs by coating PTX and DSF cationic hybrid nanoparticle cores with the vesicles. To optimize the membrane/core ratio, we prepared different CM-HNPs with increasing amounts of respective cell membrane/core ratios (w/w), ranging from 0.5 to 2 mg at fixed CBSA concentrations of 1 mg/mL. At lower concentrations (0.5, 0.75 mg) the hydrodynamic size of CM-HNPs increased, implying incomplete cell membrane coating, resulting in the exposure of nanoparticle surface to charge screening and aggregation [20]. While the membrane-to-core ratio (1 mg/1 mg) allowed for little alteration in particle size after a period of 7 days (Figure 4A–D). Notably, after the cell membrane coating, the shift of CBSA-associated positive surface charge of hybrid nanoparticles to negative confirmed the formation of a cell membrane cloaking (Figure 5A–C). In this respect, the excessively exposed carboxyl and amino groups of CBSA and electrostatic interaction between the negatively charged cell membrane vesicles and positive charge hybrid cores might be the key mechanisms of cell membrane coating. The TEM examination of A549/T CM-HNPs showed hollow and round morphology, while A549/T CM-HNPs displayed a compact inside core and a thin cell membrane coating around spherical nanoparticles (Figure 5D,E). The HNPs had encapsulation efficiency (EE) of 60%–80% for the two drugs in different formulations and a drug loading of over 22% in total, along with a PTX/DSF weight ratio of approximately 5:1 (Table 1). Additionally, the CM-HNPs have serum stability, demonstrated by the stability study that 48-incubation in PBS containing 30% serum had little influence on the diameter of NPs (Figure 6).

### 3.2. Membrane Protein Translocation and In Vitro Drug Release Kinetics 

**Figure 7:**
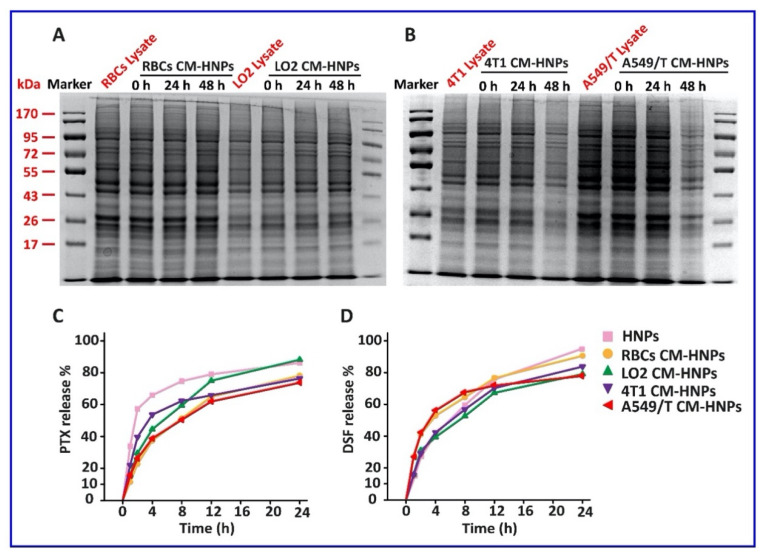
Membrane-associated protein translocation. (**A**,**B**) SDS-PAGE (Coomassie blue) assay of total membrane proteins extracted from cell membrane lysate and CM-HNPs. The in vitro release profiles of (**C**) PTX and (**D**) DSF from the nanoparticles in pH 7.4 media at 37 °C.

The aim of this study was to specifically target the A549/T cells by homotypic binding between the A549/T cells and A549/T CM-HNPs. The protein on the cell membrane is essential for the specific binding [21]. Herein, SDS-PAGE assay was used to assess whether the cell membrane-associated proteins can be translocated on the surface of CM-HNPs (Figure 7A,B). As expected, the total cell membrane protein profiles of CM-HNPs tracked at 0, 24, and 48 h after preparation were similar to that of cellular protein lysate, suggesting promising translocation. The CM-HNPs displayed a pattern of sustained drug release over 12 h (Figure 7C,D). The coating of cell membrane delayed the PTX release from the nanoparticles and had little effect on the release of DSF compared with the HNPs without coating of cell membrane, probably owing to the difference in solubility of the two drugs.

### 3.3. Homotypic Uptake Study 

**Figure 8:**
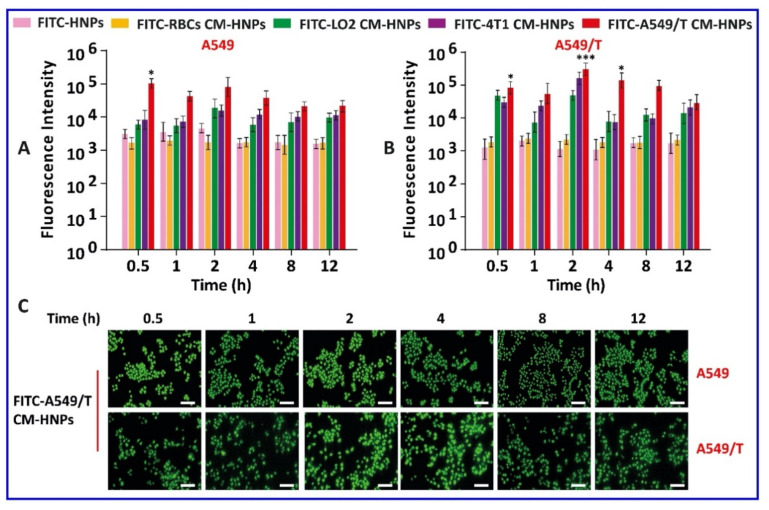
Cell uptake study. Flow cytometry analysis of cellular uptake of fluorescein 5(6)-isothiocyanate (FITC) HNPs in (**A**) A549 and (**B**) A549/T cells at a fixed FITC concentration of 50 ng/mL at 0.5, 1, 2, 4, 8, and 12 h. Microscopic images of (**C**) A549 and A549/T cells treated with FITC-A549/T CM-HNPs at a fixed FITC concentration of 50 ng/mL at 0.5, 1, 2, 4, 8, and 12 h. Scale bar: 100 μm, * *p* < 0.05 and *** *p* < 0.001 vs. FITC-HNPs.

Next, the homotypic uptake of FITC-A549/T CM-HNPs was investigated in A549 and A549/T cells. Interestingly, the FITC-A549/T CM-HNPs showed initially higher uptake in A549 cells, with maximum fluorescence intensity at 0.5 h and 2 h; after that, the fluorescence intensity decreased up to 12 h. In contrast, the fluorescence intensity in A549/T cells was initially low and was maximum at 2 h. After 2 h, the fluorescence intensity in A549/T cells was decreased gradually up to 12 h, similar to A549 cells. Noticeably, among the membrane-coated NPs, the fluorescence intensity of FITC-A549/T CM-HNPs in both A549 and A549/T cells at all time points was maximal. In particular, at 2 h, the fluorescence intensity of FITC-A549/T CM-HNPs in A549/T cells was 12-fold higher than that in A549 cells (Figure 8A,B). The fluorescence microscopic images also displayed higher green fluorescence at 2 and 4 h (Figure 8C). These results demonstrated the homotypic targeting ability. Cells basically have certain antigen receptors and proteins on their surface, which not only help them to maintain their normal physiological functions but also are responsible for inter-cellular and immunological crosstalk, whereas in cases of tumor cells, approximately 150 transmembrane proteins are overexpressed and have a potential to enhance internalization of materials [22,23,24]. Therefore, this higher uptake of A549/T CM-HNPs may be associated with the overexpressed certain membrane proteins on A549/T cells [24]. Thus, the results confirmed the efficient homotypic binding between homologous surfaces.

### 3.4. In Vitro Antitumor Effects 

**Figure 9:**
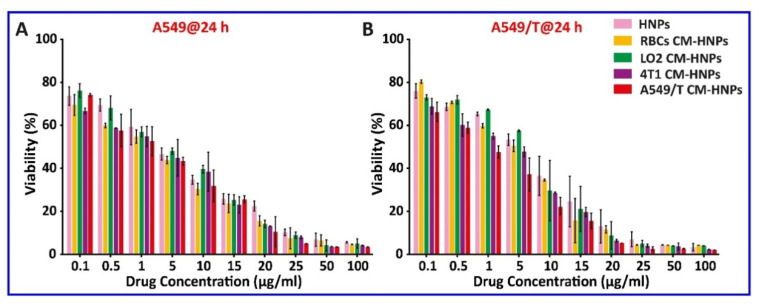
Cell viability. Viability of (**A**) A549 or (**B**) A549/T cells after treatment for 24 h at varied PTX concentrations ranging from 0.1 to 100 µg/mL; *n* = 3.

First, the antitumor activity in vitro was investigated by MTT assay. The cell viability of A549 and A549/T cells after treatment with various cell membrane-coated NPs is depicted in Figure 9A,B and Table 2. After incubation for 24 h, the IC_50_ values of HNPs, RBC CM-HNPs, LO2 CM-HNPs, 4T1 CM-HNPs, and A549/T CM-HNPs in A549 and A549/T cells were, respectively, 2.5, 1.5, 2.3, 1.8, and 1.4 µg/mL and 3.0, 2.3, 3.1, 1.7, and 1.2 µg/mL (Table 2). Notably, compared with HNPs coated with CBSA alone, the reduction in IC_50_ was 1.7- and 2.5-fold in A549 and A549/T cells, respectively. The relative resistance index (RRI) represents the extent of cellular sensitivity to drugs [25]. Intriguingly, the A549/T CM-HNPs decreased the RRI up to 1.5-fold over A549/T (Table 2), implying the suppression of P-gp.

**Figure 10:**
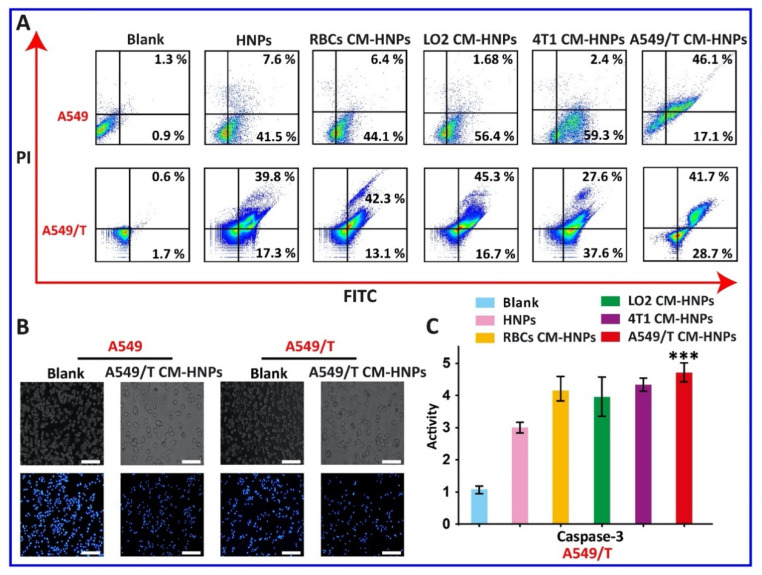
Apoptotic study. (**A**) Annexin V-FITC/PI apoptosis assay in A549 and A549/T cells after treatment with CM-HNPs at a fixed PTX concentration of 5 µg/mL for 24 h at 37 °C. (**B**) Cellular and nuclear morphology of cells after treatment. Scale bar = 100 µm. (**C**) Caspase-3 activity of A549/T cells after treatment with CM-HNPs for 24 h at 37 °C; *n* = 3, one-way ANOVA and Tukey analysis applied, and level of significance was *** *p* < 0.001 vs. blank.

To further elucidate the homotypic targeting and effect of different formulations on sensitive and MDR tumor cell viability, we performed an apoptosis assay. In A549 cells, HNPs, RBCs CM-HNPs, LO2 CM-HNPs, 4T1 CM-HNPs, and A549/T CM-HNPs induced about 49%, 50%, 58%, 61%, and 63% of apoptosis, while in A549/T cells they showed 57%, 55%, 62%, 65%, and 71%, respectively. Additionally, after treatment with A549/T CM-HNPs, both A549 and A549/T cells showed apoptotic morphology, as displayed by obvious changes in cellular and nuclear morphology, such as membrane blebbing, nuclear damage, and appearance of membrane-associated apoptotic bodies. Thus, results confirmed that the cell membrane-coated formulations efficiently induced notable apoptosis in both cell lines (Figure 10A,B). However, as compared to A549 cells, the A549/T CM-HNPs allowed for about a 1.1-fold increase of apoptosis in A549/T cells. As is well known, caspase-3 plays a critical role in inducing cell apoptosis. Therefore, to identify the results of apoptosis, the activity of caspase-3 was determined. As expected, the A549/T CM-HNPs displayed the highest caspase-3 activity among the membrane-coated NPs (Figure 10C). Overall, through the homotypic targeting-ability, the membrane-coated NPs, A549/T CM-HNPs, induced higher efficacy in apoptosis of A549/T cells.

### 3.5. P-gp Drug Efflux Inhibition and Intracellular ATP Determination 

**Figure 11:**
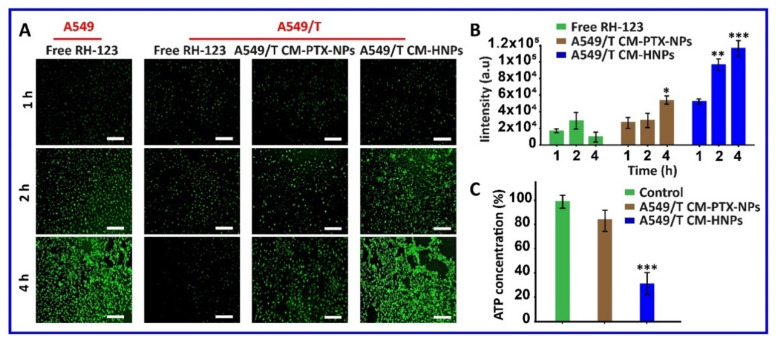
P-gp drug efflux inhibition. (**A**) Fluorescence images of A549 and A549/T cells after 1, 2, and 4 h incubation with the free Rhodamine 123 (RH-123) (5 mM) in the absence and presence of A549/T CM-HNPs and A549/T CM-PTX-NPs at a fixed PTX concentration of 5 µg/mL at 37 °C, scale bar: 100 µm. (**B**) Fluorescence intensity of A549/T cells after 1, 2, and 4 h incubation with the free RH-123 (5 mM) in the absence and presence of A549/T CM-HNPs and A549/T CM-PTX-NPs at a fixed PTX concentration of 5 µg/mL at 37 °C. (**C**) Intracellular ATP concentration in A549/T cells at 4 h post-treatment. * *p* < 0.05, ** *p* < 0.01, and *** *p* < 0.001 vs. free RH-123 and control, respectively.

The overexpressed P-gp on MDR tumor cells offers significant efflux of chemotherapeutic drugs and, as a result, discounts the treatment efficacy [26]. In view of this, we determined the enhancement of intracellular accumulation of A549/T CM-HNPs in P-gp-overexpressed A549/T cells. In this respect, Rhodamine 123 (RH-123), a P-gp substrate that is subject to recognition and secretion by the overexpressed P-gp efflux pump, was used as a model drug [27], whose cellular accumulation indicates the inhibition of P-gp and uptake of NPs. It was observed that the free RH-123 showed an enhanced fluorescence intensity within A549 cells at different time intervals but hardly entered A549/T cells, indicating a significant P-gp-mediated RH-123 efflux by A549/T cells (Figure 11A). Next, we evaluated the effect of A549/T CM-HNPs and A549/T CM-PTX-NPs (not contained DSF) on the accumulation of RH-123 within A549/T cells. The fluorescence intensity of A549/T cells after co-incubation of free RH-123, A549/T CM-HNPs, and A549/T CM -PTX-NPs revealed that the A549/T CM-HNPs markedly enhanced the fluorescence intensity of RH-123, with an increase of 3.2- and 2.1-fold at 2 and 4 h after incubation, respectively, over A549/T CM-PTX-NPs. Similarly, the microscopic images of A549/T cells at 2 and 4 h also exhibited a significant higher green fluorescence in the A549/T CM-HNPs group compared to A549/T CM-PTX-NPs (Figure 11A,B). A previous report indicated that DSF could covalently modify the cysteine residue of P-gp and inhibit its drug efflux efficiency [28]. Therefore, A549/T CM-HNPs showed higher accumulation of RH-123 in A549/T cells. Collectively, the results confirmed the DSF-associated P-gp efflux pump inactivation by A549/T CM-HNPs and the enhanced accumulation in the MDR tumor cells.

The intracellular high energy (ATP) production fuels P-gp drug efflux pump [29] and, therefore, compromises chemotherapeutic efficacy in MDR cancer cells [30]. As a result, we postulated that A549/T CM-HNPs interfered with ATP production in A549/T cells and improved the accumulation in the cells. To examine this, we incubated A549/T cells with A549/T CM-HNPs or A549/T CM-PTX-NPs for 4 h and determined the intracellular ATP-concentration. As expected, A549/T CM-HNPs inhibited the ATP production by about three- and fourfold in A549/T cells as compared to A549/T CM-PTX-NPs and control, respectively (Figure 11C). These data confirmed that (i) A549/T CM-HNPs efficiently targeted to MDR tumor cells, enabling enhanced delivery of both PTX and DSF, (ii) the A549/T CM-HNPs significantly downregulated the level of ATP concentration, and therefore suppressed the P-gp drug efflux pump [31] and sensitized the MDR cells to cytotoxic PTX [32]. 

### 3.6. Hemotoxicity Assay

**Figure 12:**
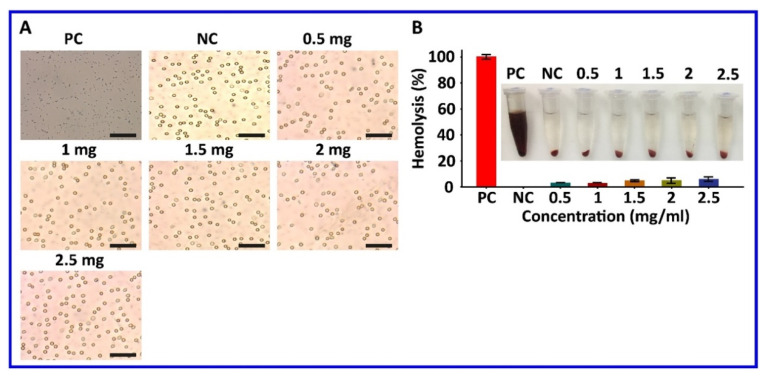
Hemotoxicity assay. (**A**) Microscopic examination of RBC membrane damage at 2 h post-incubation with A549/T CM-HNPs at various concentrations (0.5–2.5 mg/mL). (**B**) Quantitative estimation of RBC membrane damage and hemoglobulin release at 2 h post-incubation. Triton-X (20%) and saline were used as positive and negative controls (PC and NC), respectively.

A critical safety concern of blood-contacting nanomedicines is their hemocompatibility [33]. In the study, Triton-X 100 (PC control) exacerbated the hemolysis and induced complete RBC membrane damage; on the other hand, no hemolysis was observed in saline-treated RBCs (NC control) (Figure 12A). Notably, treatment with A549/T CM-HNPs at all tested concentrations induced little hemolysis. Morphological examination after 2 h incubation further confirmed no significant RBC membrane damage (Figure 12B), which could be associated with natural biocompatibility of cell membranes, on coated nanoparticles [34]. These data suggested A549/T CM-HNPs are safe for in vivo testing.

## 4. Conclusions

In summary, the coating of nanoparticles with a specific cell membrane provides an effective strategy of surface functionalization for self-recognition and targeting. In this report, we demonstrated the isolation of various cell membranes with intact membrane proteins and their successful cloaking on cationic hybrid nanoparticle core (≥200 nm) to target MDR tumor cells. The A549/T CM-HNPs significantly inhibited the P-gp drug efflux pump by decreasing the ATP concentration and, as a result, improved the cellular uptake of the drugs and potently induced caspase-3-associated apoptosis in MDR tumor cells. Most importantly, this study offered proof-of-concept of active-targeting of homologous MDR cancer cell line by coating NPs with homotypic membrane. Overall, cell membrane coating based on “homotypic binding” is a promising route to improve the accumulation of chemotherapeutic drugs in MDR cells and kill them.

## Figures and Tables

**Table 1:** Drug loading (DL) and encapsulation efficiency (EE) of various formulations.

	PTX		DSF	
Formulations	DL	EE	DL	EE
HNPs	23.1 ± 1.1	65.4 ± 1.6	4.9 ± 1.5	84.3 ± 3.7
RBCs CM-HNPs	26.7 ± 1.2	71.2 ± 0.4	6.2 ± 0.6	86.7 ± 1.8
LO2 CM-HNPs	19.6 ± 2.9	63.6 ± 2.1	3.4 ± 0.4	79.8 ± 3.2
4T1 CM-HNPs	18.4 ± 0.7	66.8 ± 3.6	3.8 ± 1.9	72.6 ± 2.4
A549/T CM-HNPs	24.8 ± 3.5	69.4 ± 3.1	5.4 ± 1.2	71.4 ± 3.6

**Table 2:** Cytotoxicity of different formulation in A549 and A549/T cells after 24 h of incubation.

Formulation	IC_50_^a^ (µg)		RRI ^b^
	A549@24 h	A549/T@24 h	24 h
HNPs	2.5 ± 0.7	3.0 ± 0.5	1.2
RBCs CM-HNPs	1.5 ± 0.3	2.3 ± 0.1	1.5
LO2 CM-HNPs	2.3 ± 0.6	3.1 ± 0.8	1.3
4T1 CM-HNPs	1.8 ± 0.7	1.7 ± 0.4	0.9
A549/T CM-HNPs	1.4 ± 0.6 *	1.2 ± 0.1 **	0.8

^a^ Dose required to inhibit 50% of cell growth. ^b^ Relative resistance index (RRI): IC_50_ of A549/T divided by the IC_50_ of A549, paired *t*-test was applied, and level of significance was * *p* < 0.05 and ** *p* < 0.001 vs. HNPs.
